# 

**Published:** 1918-03

**Authors:** 


### ANNOUNCEMENTS

AMERICAN INSTITUTE OF DENTAL TEACHERS. At the last annual meeting of the American Institute of Dental Teachers, held at Pittsburgh, Penn., January 29-31, 1918, the following officers were elected; President, Dr. A. W. Thornton, McGill University, Department of Dentistry, Montreal, Quebec; Vice-President, Dr. R. W. Bunting, Ann Arbor, Mich.; Secretary-Treasurer, Dr. Abram Hoffman, 381 Linwood Avenue, Buffalo, N. Y.; Executive Board, Dr. A. D. Black, Chicago, Ill., Dr. G. S. Millberry, San Francisco, Cal., and Dr. A. H. Hipple, Omaha, Neb.

The next annual meeting will be held January 28, 29 and 30, 1919. The place of meeting will be announced later.

THE RESEARCH INSTITUTE OF THE NATIONAL DENTAL ASSOCIATION. The present status is briefly as follows:

The support of the Institute has been substantially enlarged. Three-fourths of the membership of the National Dental Association has now adopted the one dollar dues plan for the support of the Research Institute, and is providing, from this source, an income of $15,000 annually. This is being steadily increased and takes the place of the expiring pledges.

The Building and Equipment Fund is steadily increasing. The second mortgage has been reduced to $4,500. The first mortgage is $27,000. The pledges in hand are about $7,000, making the balance to be provided for the building and equipment about $25,000. The equipment has been increased to $10,430 by purchases and gifts, about $6,000 of which has been in donations of required apparatus and equipment, largely from dental manufacturers. The net worth of the Institute, as found by the audit of the authorized accountants June 20, was $69,317.

A skilled research director is at this time being engaged to give his entire time to the work of the Institute. He will devote his immediate energies largely to the problems of the army, and particularly to the study of trench mouth.

The following important actions were taken by the National Dental Association at the recent New York meeting:

The House of Delegates adopted, to become a part of the Constitution and By-laws, the following:

"That state and constituent societies add one dollar to their dues for the support of the Research Institute, and the funds so collected shall be remitted directly to the Research Institute, and that the treasurer of the Research Institute report to the House of Delegates those states and constituent societies complying with this request."

The joint meeting of the trustees of the National Dental Association, the trustees of the Research Institute and the members of the Research Commission adopted the following policies for the Institute:

That the researches under grants be continued; that the research work in the headquarters be continued; that a research director be engaged as soon as practicable to devote his entire time to the scientific work in the headquarters, with skilled assistants; that the problems in the Institute be limited to pathological problems.

It is expected that the research director will be selected soon to take charge of the staff and the supervision of the scientific work. This duty has been temporarily carried, until the Institute could afford to engage a research director, by the acting managing director. The available energy of the Institute has been directed, during the past year, to adding to our exact knowledge regarding the management of pulpless teeth. These researches were reported at the national meeting in New York in October, and a summary of the results will be reviewed in the seminary and clinics at this meeting.

The profession in Ohio has furnished approximately twenty per cent. of the money for carrying on the researches under grant throughout the United States and in the Institute since the organization of the department, and approximately forty per cent. of the Building and Equipment Fund. It is quite just, therefore, that they should have some of the results of this work presented to them directly.

WESTON A. PRICE, Director.

